# Effect of exposure to radiation caused by an atomic bomb on endothelial function in atomic bomb survivors

**DOI:** 10.3389/fcvm.2023.1122794

**Published:** 2023-02-17

**Authors:** Shinji Kishimoto, Nozomu Oda, Tatsuya Maruhashi, Shunsuke Tanigawa, Aya Mizobuchi, Farina Mohamad Yusoff, Asuka Fujita, Toshio Uchiki, Masato Kajikawa, Kenichi Yoshimura, Takayuki Yamaji, Takahiro Harada, Yu Hashimoto, Yukiko Nakano, Seiko Hirota, Shinji Yoshinaga, Chikara Goto, Ayumu Nakashima, Yukihito Higashi

**Affiliations:** ^1^Department of Regenerative Medicine, Research Institute for Radiation Biology and Medicine, Hiroshima University, Hiroshima, Japan; ^2^Department of Cardiovascular Medicine, Hiroshima Red Cross Hospital and Atomic-Bomb Survivors Hospital, Hiroshima, Japan; ^3^Plastic and Reconstructive Surgery, Hiroshima University Hospital, Hiroshima, Japan; ^4^Division of Regeneration and Medicine, Medical Center for Translational and Clinical Research, Hiroshima University Hospital, Hiroshima, Japan; ^5^Department of Cardiovascular Medicine, Graduate School of Biomedical and Health Sciences, Hiroshima University, Hiroshima, Japan; ^6^Department of Environmetrics and Biometrics, Research Institute for Radiation Biology and Medicine, Hiroshima University, Hiroshima, Japan; ^7^Department of Rehabilitation, Faculty of General Rehabilitation, Hiroshima International University, Hiroshima, Japan; ^8^Department of Stem Cell Biology and Medicine, Graduate School of Biomedical and Health Sciences, Hiroshima University, Hiroshima, Japan

**Keywords:** radiation exposure, endothelial function, arterial stiffness, atomic bomb survivors, flow-mediated vasodilation (FMD)

## Abstract

**Background:**

The purpose of this study was to evaluate the effects of exposure to radiation caused by an atomic bomb in atomic bomb survivors on vascular function and vascular structure and to evaluate the relationships of radiation dose from the atomic bomb with vascular function and vascular structure in atomic bomb survivors.

**Methods:**

Flow-mediated vasodilation (FMD) and nitroglycerine-induced vasodilation (NID) as indices of vascular function, brachial-ankle pulse wave velocity (baPWV) as an index of vascular function and vascular structure, and brachial artery intima-media thickness (IMT) as an index of vascular structure were measured in 131 atomic bomb survivors and 1,153 control subjects who were not exposed to the atomic bomb. Ten of the 131 atomic bomb survivors with estimated radiation dose in a cohort study of Atomic Bomb Survivors in Hiroshima were enrolled in the study to evaluate the relationships of radiation dose from the atomic bomb with vascular function and vascular structure.

**Results:**

There was no significant difference in FMD, NID, baPWV, or brachial artery IMT between control subjects and atomic bomb survivors. After adjustment of confounding factors, there was still no significant difference in FMD, NID, baPWV, or brachial artery IMT between control subjects and atomic bomb survivors. Radiation dose from the atomic bomb was negatively correlated with FMD (ρ = −0.73, *P* = 0.02), whereas radiation dose was not correlated with NID, baPWV or brachial artery IMT.

**Conclusion:**

There were no significant differences in vascular function and vascular structure between control subjects and atomic bomb survivors. Radiation dose from the atomic bomb might be negatively correlated with endothelial function.

## Introduction

It is well-known that high-dose radiation increases the risk of cardiovascular disease (1), whereas the relationship between low-dose or middle-dose radiation and risk of cardiovascular disease is controversial (1–4). Shimizu et al. (1) showed that a dose of more than 0.5 Gy was correlated with an increased risk of cardiovascular disease but that there was no relationship between a radiation dose of less than 0.5 Gy and cardiovascular disease in atomic bomb survivors who had been followed up for 53 years. Tran et al. (4) showed that a radiation dose of less than 0.5 Gy was associated with mortality of cardiovascular disease in patients with tuberculosis. Some studies have shown that atomic bomb survivors have persistent inflammation that is positively correlated with radiation dose from the atomic bomb (5, 6). Inflammation plays a crucial role in the progression of atherosclerosis (7). Long-term observational studies in atomic bomb survivors showed that radiation to which they were exposed from the atomic bomb is associated with increased blood pressure, serum cholesterol level and incidence of diabetes, which are risk factors for atherosclerosis (8–10). While radiation therapy has improved the prognosis of cancer patients, cardiac exposure from radiation therapy causes an increase in the risk of coronary heart disease, which continues for a long time (11, 12). In addition, long-term low-dose radiation exposure in healthcare workers was shown to be associated with early atherosclerosis (13). Therefore, it is important to know the long-term effects of radiation from an atomic bomb on the vasculature function.

It is recognized that endothelial dysfunction occurs from the early stages of atherosclerosis development. Endothelial dysfunction plays an important role in cardiovascular complications (14, 15). Flow-mediated vasodilation (FMD) was measured as an indicator of endothelial function, and nitroglycerine-induced vasodilation (NID) was measured as an indicator of vascular smooth muscle function (16, 17). Several investigators have reported that vascular dysfunction predicts cardiovascular events (18, 19). Measurement of brachial-ankle pulse wave velocity (baPWV) as an index of arterial stiffness, which is an indicator of both vascular function and vascular structure, and measurement of brachial artery intima-media thickness (IMT) as an index of vascular structure have been shown to be significantly correlated with cardiovascular risk factors (20, 21).

The purpose of this study was to evaluate the effects of radiation caused by the atomic bomb in atomic bomb survivors 65 years or longer after exposure to the radiation on vascular function and vascular structure and to evaluate the relationships of the dose of radiation from the atomic bomb to which the survivors were exposed with vascular function and vascular structure in atomic bomb survivors.

## Materials and methods

### Study protocol 1: Vascular function in atomic bomb survivors

Between August 2010 and June 2021, a total of 131 atomic bomb survivors were recruited for vascular function measurement from subjects who attended the outpatient clinic at Hiroshima University Hospital, and 3,966 control subjects who were not exposed to the atomic bomb were recruited from the Hiroshima University Hospital Vascular Registry. All of the atomic bomb survivors who participated in the study consented to the measurement and study participation, and those who did not consent were excluded. An atomic bomb survivor was defined as an individual who was formally issued an Atomic Bomb Health Handbook based on the law concerning assistance for atomic bomb survivors and who met one or more of the following conditions: having been directly exposed within a few kilometers of the hypocenter of the atomic bomb, having entered within two kilometers of the hypocenter within 2 weeks after the bombing, having been engaged in rescue or other related activities, and having been exposed *in utero*. The cohort of Atomic Bomb Survivors in Hiroshima (ABS) included about 290,000 atomic bomb survivors in Hiroshima who were issued an Atomic Bomb Health Handbook. ABS was a cohort study that was started in 1971 by the Research Institute for Radiation Biology and Medicine of Hiroshima University. Details of the ABS study methods were described previously (22). Of the 3,966 control subjects, 2,813 subjects who were less than 65 years old were excluded because this study was started 65 years after the atomic bombing and the youngest age of the atomic bomb survivors was 65 years. Finally, 1,153 control subjects were enrolled in this study. Diabetes mellitus was defined according to the criteria provided by the American Diabetes Association or a previous diagnosis of diabetes (23, 24). The definition of dyslipidemia was based on the third report of the National Cholesterol Education Program (25).

All measurements were done in the morning, after overnight fasting, in a quiet, dark, air-conditioned room (constant temperature of 22–25°C) during the study. FMD, NID, baPWV, and brachial artery IMT were measured, after maintaining the supine position for 30 min. The observers masked the clinical characteristics of the subjects and the aim of the study. All methods were carried out according with the Declaration of Helsinki and relevant guidelines and regulations. The study was approved by the Ethics Review Board of Hiroshima University. Written informed consent was obtained from all subjects.

### Study protocol 2: Relationship between vascular function and dose of radiation from the atomic bomb to hich atomic bomb survivors were exposed

After checking all members of Protocol 1 against the ABS cohort, 121 of the 131 atomic bomb survivors without estimated dose of radiation to which they were exposed from the atomic bomb in a cohort study of ABS in Hiroshima were excluded. Finally, 10 atomic bomb survivors whose radiation dose was accurately assessed were enrolled in study protocol 2.

### Measurements of FMD and NID

Flow-mediated vasodilation was measured as endothelium-dependent vasodilation of vascular response to reactive hyperemia in the brachial artery by using an automated edge detection system (UNEXEF18G, UNEX Co, Nagoya, Japan) (26). NID was measured in vascular response to nitroglycerine as endothelium-independent vasodilation, as previously reported (26). This study was conducted with a methodological approach to FMD, following the recommendations proposed by Thijssen et al. (27). Additional details can be found in the Supplementary material.

### Measurement of brachial IMT and baPWV

Details on the measurement of Brachial IMT and baPWV can be found in the Supplementary material.

### Radiation dosimetry

We used the dose of exposure to radiation from neutrons and gamma rays estimated by using the Atomic Bomb Survivor 1993 Dose (ABS93D). ABS93D was described in detail in a previous report (28). Briefly, radiation dose calculated by ABS93D is based on individual exposure status such as distance from the hypocenter, shielding and age at time of bombing. Hoshi et al. (28) showed that the dose evaluation of ABS93D was close to that of the Dosimetry system 1986 (DS86) by Radiation Effects Research Foundation. We used the weighted radiation dose of the colon, which is often chosen as the whole-body irradiation exemplary organ, by calculating the sum of the gamma ray dose and 10 times the neutron dose considering the biological effectiveness of neutrons.

### Statistical analysis

Results are summarized as means ± SD for continuous variables and as percentages for categorical variables. A 2-sided probability value of <0.05 was considered to indicate statistical significance. The FMD value in subjects over 60 years old was determined to be 2.7 ± 2.5% in a previous study (29). The number of subjects needed to detect a difference of 1.0% FMD and a standard deviation (SD) of 2.5% between two groups with a probability of 0.05 and a power of 0.80 was 100 per group. Continuous variables were compared by using ANOVA. Categorical variables were compared by using chi-square test. Relationships between variables were determined using Spearman’s correlation coefficients. To create a matched cohort of control subjects and atomic bomb survivors, a propensity score was calculated using logistic regression analysis of the probability of baseline clinical variables in two models: model 1 including age and sex and model 2 including age, sex, body mass index, heart rate, hypertension, dyslipidemia, diabetes mellitus, and current smokers. To create matched pairs to investigate the associations of exposure to radiation with vascular function and vascular structure, matched pairs were using one-to-one propensity-score matching analyses. The caliper size of propensity scores was used a quarter of a standard deviation of the sample estimated propensity scores for comparison of vascular function. The data were processed using JMP pro version 15 (SAS Institute, Cary, NC, USA).

## Results

### Study protocol 1: Baseline clinical characteristics

The baseline clinical characteristics of the 1,153 control subjects and 131 atomic bomb survivors are summarized in Table 1. There were significant differences in sex, heart rate and estimated glomerular filtration rate between control subjects and atomic bomb survivors.

**TABLE 1:** Clinical characteristics of the subjects corresponding to study protocol 1.

Variables	Control subjects (*n* = 1,153)	Atomic bomb survivors (*n* = 131)	*P*-value
Age, year	76 ± 5	76 ± 5	0.92
Age at atomic bomb exposure, year		5 ± 4	
Sex, men/women	623/530	92/39	<0.01
Body mass index, kg/m^2^	23.0 ± 3.4	23.5 ± 3.0	0.09
Systolic blood pressure, mmHg	130 ± 18	128 ± 19	0.21
Diastolic blood pressure, mmHg	74 ± 11	73 ± 11	0.26
Heart rate, bpm	70 ± 12	66 ± 11	<0.01
Total cholesterol, mg/dL	184 ± 36	178 ± 36	0.07
Triglycerides, mg/dL	122 ± 70	120 ± 70	0.80
HDL cholesterol, mg/dL	60 ± 17	59 ± 18	0.62
LDL cholesterol, mg/dL	104 ± 30	99 ± 29	0.14
Glucose, mg/dL	118 ± 38	113 ± 27	0.24
HbA1c, %	6.1 ± 0.8	6.2 ± 0.8	0.22
eGFR, mL/min per 1.73 m^2^	60.1 ± 18.8	56.0 ± 18.8	0.03
High-sensitivity CRP, mg/dL	0.46 ± 1.12	0.59 ± 1.32	0.58
**Medical history, n (%)**			
Hypertension	927 (80.4)	102 (77.9)	0.50
Dyslipidemia	794 (68.9)	81 (61.8)	0.11
Diabetes mellitus	442 (38.3)	49 (37.4)	0.84
Previous coronary heart disease	219 (19.0)	34 (26.0)	0.07
Previous stroke	128 (11.1)	17 (13.0)	0.54
Current smoker	87 (7.6)	10 (7.6)	0.97
**Medication, n (%)**			
Antihypertensive drugs	901 (78.1)	107 (81.7)	0.34
Lipid-lowering drugs	629 (40.6)	72 (55.0)	0.93
Antidiabetic drugs	341 (29.6)	36 (27.5)	0.62

HDL, high-density lipoprotein; LDL, low-density lipoprotein; HbA1c, hemoglobin A1c; eGFR, estimated glomerular filtration rate; CRP, C-reactive protein. Results are presented as means ± SD for continuous variables and percentages for categorical variables.

Moreover, we assessed vascular function in atomic bomb survivors using propensity score matching to create matched pairs between control subjects and atomic bomb survivors. In propensity score-matched pairs of control subjects and atomic bomb survivors in model 1, the clinical characteristics of matched pairs of 131 control subjects and 131 atomic bomb survivors are summarized in Supplementary Table 1. In propensity score-matched pairs of control subjects and atomic bomb survivors in model 2, the clinical characteristics of matched pairs of 119 control subjects and 119 atomic bomb survivors are summarized in Supplementary Table 2.

### Study protocol 1: Vascular function and vascular structure in atomic bomb survivors

**FIGURE 1:**
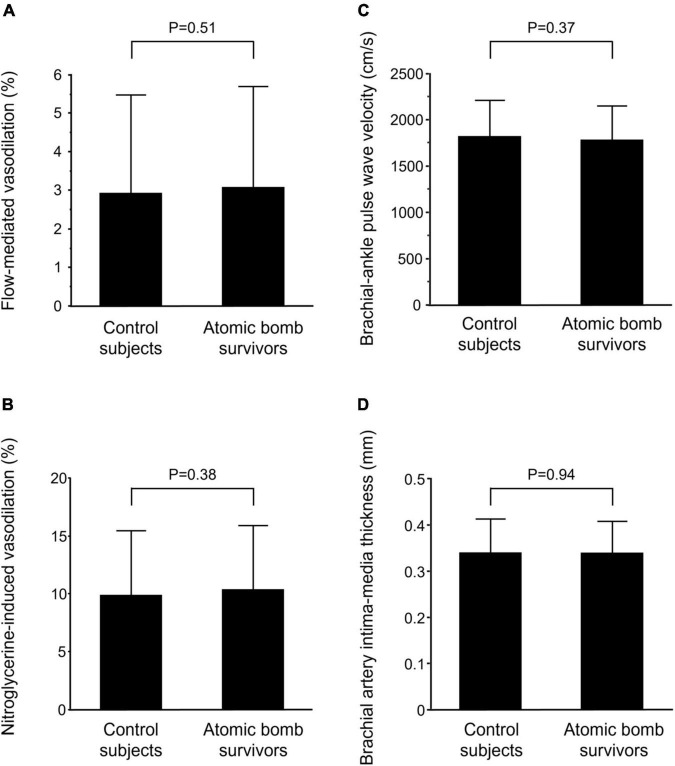
Bar graphs show flow-mediated vasodilation **(A)**, nitroglycerine-induced vasodilation **(B)**, brachial-ankle pulse wave velocity **(C)**, and brachial artery intima-media thickness **(D)** in control subjects and atomic bomb survivors.

There was no significant difference in FMD, NID, baPWV, or brachial artery IMT between control subjects and atomic bomb survivors (2.9 ± 2.6 vs. 3.1 ± 2.6%, 9.9 ± 5.6 vs. 10.4 ± 5.5%, 1819 ± 391 vs. 1782 ± 366 cm/s, and 0.34 ± 0.07 vs. 0.34 ± 0.07 mm, *P* = 0.51, *P* = 0.38, *P* = 0.37, and *P* = 0.94, respectively) (Figure 1).

In propensity score-matched pairs of control subjects and atomic bomb survivors in model 1, there was no significant difference in FMD, NID, baPWV, or brachial artery IMT between control subjects and atomic bomb survivors (2.8 ± 2.4 vs. 3.1 ± 2.6%, 10.5 ± 5.8 vs. 10.4 ± 5.5%, 1853 ± 379 vs. 1782 ± 366 cm/s, and 0.35 ± 0.07 vs. 0.34 ± 0.07 mm, *P* = 0.44, *P* = 0.90, *P* = 0.16, and *P* = 0.16, respectively) (Supplementary Figure 1). In propensity score-matched pairs of control subjects and atomic bomb survivors in model 2, there was no significant difference in FMD, NID, baPWV, or brachial artery IMT between control subjects and atomic bomb survivors (2.6 ± 2.3 vs. 3.1 ± 2.7%, 9.5 ± 5.6 vs. 10.5 ± 5.6%, 1753 ± 358 vs. 1783 ± 354 cm/s, and 0.34 ± 0.07 vs. 0.34 ± 0.06 mm *P* = 0.11, *P* = 0.19, *P* = 0.57, and *P* = 0.71, respectively) (Supplementary Figure 2).

### Study protocol 2: Baseline clinical characteristics of atomic bomb survivors whose radiation dose was accurately assessed

The baseline clinical characteristics of the 10 atomic bomb survivors who radiation dose was accurately assessed are summarized in Supplementary Table 3. Of the 10 atomic bomb survivors, nine (90.0%) had hypertension, eight (80.0%) had dyslipidemia, three (30.0%) had diabetes mellitus, four (40.0%) had previous coronary heart disease, three (30.0%) had previous stroke and one (10.0%) was a current smoker. Mean values were 3.9 ± 1.5% for FMD, 11.8 ± 4.4% for NID, 1630 ± 234 cm/s for baPWV, and 0.32 ± 0.03 mm for brachial artery IMT.

### Study protocol 2: Effects of radiation dose on vascular function and vascular structure in atomic bomb survivors whose radiation dose was accurately assessed

**FIGURE 2:**
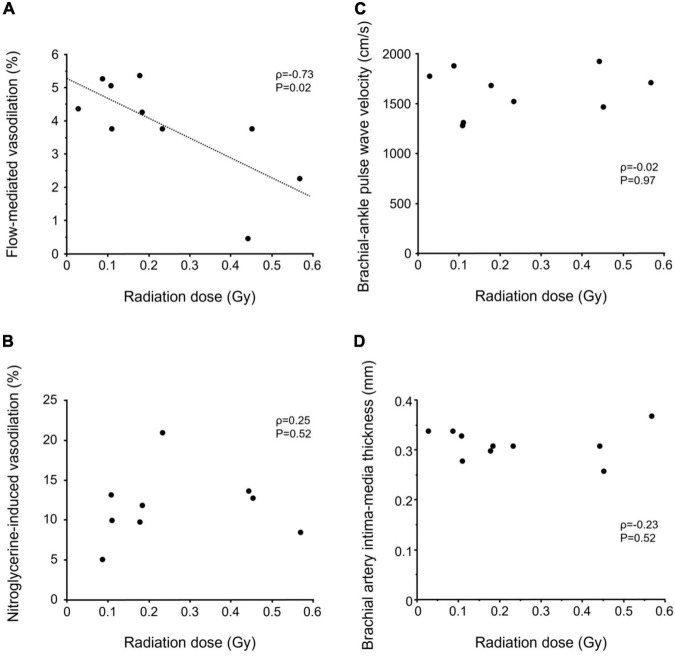
Scatter plots show relationships of radiation dose with flow-mediated vasodilation **(A)**, nitroglycerine-induced vasodilation **(B)**, brachial-ankle pulse wave velocity **(C)**, and brachial artery intima-media thickness **(D)** in atomic bomb survivors.

Radiation dose from the atomic bomb was negatively correlated with FMD (ρ = −0.73, *P* = 0.02) (Figure 2A), whereas radiation dose was not correlated with NID, baPWV, or brachial artery IMT (ρ = 0.25, *P* = 0.52; ρ = −0.02, *P* = 0.97; and ρ = −0.23, *P* = 0.52; respectively) (Figures 2B–D).

## Discussion

In the present study, we demonstrated that there was no significant difference in FMD, NID, baPWV, or brachial artery IMT between control subjects and atomic bomb survivors even after adjustment for confounding factors. Radiation dose from the atomic bomb was negatively correlated with FMD, whereas radiation dose was not correlated with NID, baPWV, or brachial artery IMT. As far as we know, this is the first study to assess vascular function and vascular structure in atomic bomb survivors 65 years or longer after exposure to radiation from the atomic bomb.

Previous studies showed a relationship between radiation exposure and cardiovascular disease in atomic bomb survivors (1–4). Shimizu et al. (1) showed that a dose of more than 0.5 Gy was correlated with an increased risk of cardiovascular disease but that there was no relationship between a dose of less than 0.5 Gy and cardiovascular disease in atomic bomb survivors who had been followed up for 53 years. Nakamizo et al. (30) showed that low-dose or mild-dose radiation exposure increased aorta calcification measured by X-ray films and carotid artery plaque measured by carotid ultrasound, whereas there were no significant relationship of dose of radiation exposed by the atomic bomb with augmentation index or baPWV in atomic bomb survivors examined from 2010 to 2014. However, there has been no information on vascular function in atomic bomb survivors 65 years or longer after radiation exposure. In the present study, there were no significant differences in vascular function and arterial structure between control subjects and atomic bomb survivors. We also performed analyses using two models of adjustment for cardiovascular risk factors. In both adjustment models, there were no significant differences in vascular function and vascular structure between control subjects and atomic bomb survivors. These findings suggest that radiation exposed by the atomic bomb has no specific effects on vascular function and vascular structure in atomic bomb survivors 65 years or longer after exposure to radiation from the atomic bomb.

It is well-known that high-dose radiation increases the risk of cardiovascular disease, whereas the relationship between low-dose or middle-dose radiation and the risk of cardiovascular disease is controversial (1–4). Tran et al. (4) showed that a dose of less than 0.5 Gy was associated with mortality of cardiovascular disease in patients with tuberculosis. In the present study, endothelial function did not differ between atomic bomb survivors and control subjects. Atomic bomb survivors were over 75 years of age and had several cardiovascular risk factors other than aging. Those risk factors may mask the effects of low to medium doses of radiation on vascular function. Interestingly, the dose of radiation exposed to was negatively correlated with FMD in atomic bomb survivors who were exposed to a dose of less than 0.6 Gy, suggesting that endothelial function might be impaired in relation to dose-response within the range of low to medium doses of radiation.

The pathogenesis of cardiovascular disease due to a high radiation dose is damage to endothelial cells and inflammation (5, 31), although the pathogenesis of cardiovascular disease due to low-dose or middle-dose radiation is unclear. In an *in vitro* study, Cervelli et al. (32) showed that exposure of human umbilical vein endothelial cells to single doses of less than 0.5 Gy resulted in increased intercellular adhesion molecule-1 and oxygen species generation. Mitchel et al. (33) showed that exposure to single doses of 0.025–0.5 Gy decreased the frequency of atherosclerosis lesion in ApoE^–/–^ mice, whereas those doses of radiation increased inflammation, total serum cholesterol levels and severity of the atherosclerosis lesions. On the other hand, some investigators showed that low-dose radiation has an anti-inflammatory effect (34, 35). Although those *in vitro* and *in vivo* studies showed short-term effects of radiation on the vasculature, the long-term effects of radiation on the vasculature are unclear. Long-term observational studies for atomic bomb survivors showed that the dose of radiation exposed by the atomic bomb was associated with increases in blood pressure, serum cholesterol level and incidence of diabetes, which are risk factors for atherosclerosis (8–10). Hayashi et al. (36) showed that atomic bomb survivors have persistent inflammation that is positively correlated with to the dose of radiation exposed by the atomic bomb. Kusunoki et al. (6) showed that T-cell immunity was attenuated in atomic bomb survivors and that there was a significant negative correlation between inflammatory markers and the number of naïve CD4 T cells. These findings suggest that T-cell immunosenescence may be partly responsible for the prolonged inflammation in atomic bomb survivors. In the present study, a radiation dose of less than 0.6 Gy was negatively correlated with FMD in atomic bomb survivors 65 years or longer after radiation exposure. A low or medium dose of radiation may impair endothelial function *via* immunological effects, inflammation, and metabolic changes in relation to radiation dose.

This study has some limitations. First, the number of atomic bomb survivors whose radiation dose was accurately assessed was relatively small. The reason for the lack of exposure data for atomic bomb survivors was that it was not known whether they entered within 2 km of the hypocenter after the atomic bomb explosion or the exact location of the exposure was not known. ABS93D provided dose estimates for 33,173 individuals since dose estimates for atomic bomb survivors by ABS93D were only data for direct exposure. Those individuals accounted for only 11.4% of the total population of the ABS cohort. After checking all members of Protocol 1 against the ABS cohort, only 10 atomic bomb survivors were provided dose estimates. Those 10 atomic bomb survivors accounted for 7.6% of the total population of Protocol 1. The proportion of atomic bomb survivors with dose estimates in the present study was slightly less than that of the overall ABS cohort. The present study may have included a large percentage of atomic bomb survivors who entered an area within 2 km of the hypocenter less than 2 weeks after the bombing, who were engaged in rescue or other related activities, or who had been directly exposed without the exact location of the exposure known. However, we found that the dose of radiation exposed by the atomic bomb was negatively correlated with FMD even in a small number of subjects. In the present study, the estimated glomerular filtration rate in atomic bomb survivors was significantly lower than that in control subjects. Sera et al. (37) showed that chronic kidney disease was significantly associated with radiation dose in atomic bomb survivors. The subjects in Study Protocol 1 might be a good representation of the overall ABS cohort. Second, atomic bomb survivors have received free medical care and may have received earlier detection and treatment of cardiovascular disease than subjects in the control group under the government care system. Further studies are needed to confirm the results of this study in trials by adjusting for the duration of cardiovascular diseases and treatment duration. Third, in the present study, biochemical oxidative stress markers and inflammatory markers were not measured in all of the subjects. Measurements of biochemical oxidative stress markers and inflammatory markers would allow more specific conclusions to be drown concerning the effect of radiation on vascular function. Fourth, the present study may have some selection bias. Atomic bomb survivors who came to the outpatient clinic at Hiroshima University Hospital were included, but those who were too frail to come to the outpatient clinic or had severe cognitive dysfunction that prevented them from consenting to this study were excluded. It is possible that only atomic bomb survivors in good physical or/and cognitive condition were included in this study. Therefore, the results of this study can be adapted to subjects who maintain activities of daily living that allow them to visit an outpatient clinic. Fifth, assessment of vascular structure in the present study by using brachial IMT and the use of small vessels may have made it difficult to detect differences. However, there was no significant difference in baPWV, as an index of vascular function and vascular structure. These findings suggest that there was no significant difference in vascular structure.

## Conclusion

There was no significant difference in vascular function or vascular structure between control subjects and atomic bomb survivors even after adjustment for cardiovascular risk factors. The data suggested that endothelial function might decrease in relation to increase in radiation dose within a relatively low range of doses of radiation from the atomic bomb. Further studies are needed to confirm the findings of this study in a large and longitudinal trial.

## Data availability statement

The raw data supporting the conclusions of this article will be made available by the authors, without undue reservation.

## Ethics statement

The studies involving human participants were reviewed and approved by the Ethics Review Board of Hiroshima University. The patients/participants provided their written informed consent to participate in this study.

## Author contributions

SK and YHi contributed to the study design and writing of the manuscript. SK, NO, TM, ST, AM, FY, AF, TU, MK, TY, TH, YHa, YN, SH, SY, CG, and AN performed the data collection. SK and KY performed statistical analyses after discussion with all authors. YN revised the manuscript critically for important intellectual content. All authors contributed to interpretation of data and review of the manuscript and read and agreed to the published version of the manuscript.
