# Supplementary material for: Effect of exposure to radiation caused by an atomic bomb on endothelial function in atomic bomb survivors
Source: Front Cardiovasc Med. 2023 Feb 17;10:1122794. doi: 10.3389/fcvm.2023.1122794 (PMC9981625; doi:10.3389/fcvm.2023.1122794)
Supplement: Supplementary file 1 [file Data_Sheet_1.docx]

Supplementary Material

Effect of Exposure to Radiation Caused by An Atomic Bomb on Endothelial Function in Atomic Bomb Survivors

Shinji Kishimoto, MD, PhD^1^, Nozomu Oda, MD, PhD^2^, Tatsuya Maruhashi, MD, PhD^1^, Shunsuke Tanigawa, MS^1^, Aya Mizobuchi, MS^1^, Farina Mohamad Yusoff, MD, PhD^1^, Asuka Fujita, MD^1,3^, Toshio Uchiki, MD^1,3^, Masato Kajikawa, MD, PhD^4^, Kenichi Yoshimura, PhD^4^, Takayuki Yamaji, MD, PhD^5^, Takahiro Harada, MD, PhD^5^, Yu Hashimoto, MD^5^, Yukiko Nakano, MD, PhD^5^, Seiko Hirota, MS^6^, Shinji Yoshinaga, PhD^6^, Chikara Goto, PhD^7^, Ayumu Nakashima, MD, PhD^8^, Yukihito Higashi, MD, PhD, FAHA^1,4^

^1^Department of Regenerative Medicine, Research Institute for Radiation Biology and Medicine, Hiroshima University, Hiroshima, Japan

^2^Department of Cardiovascular Medicine, Hiroshima Red Cross Hospital and Atomic-bomb Survivors Hospital, Hiroshima, Japan

^3^Plastic and Reconstructive Surgery, Hiroshima University Hospital, Hiroshima, Japan

^4^Division of Regeneration and Medicine, Medical Center for Translational and Clinical Research, Hiroshima University Hospital, Hiroshima, Japan

^5^Department of Cardiovascular Medicine, Graduate School of Biomedical and Health Sciences, Hiroshima University, Hiroshima, Japan

^6^Department of Environmetrics and Biometrics, Research Institute for Radiation Biology and Medicine, Hiroshima University, Hiroshima, Japan

^7^Dpartment of Rehabilitation, Faculty of General Rehabilitation, Hiroshima International University, Hiroshima, Japan

^8^Department of Stem Cell Biology and Medicine, Graduate School of Biomedical and Health Sciences, Hiroshima University Hiroshima, Japan

*** Correspondence:**Yukihito Higashi, MD, PhD, FAHA
Department of Regenerative Medicine,
Research Institute for Radiation Biology and Medicine, Hiroshima University
1-2-3 Kasumi, Minami-ku, Hiroshima 734-8551, Japan
Phone: +81-82-257-5831 Fax: +81-82-257-5831
E-mail: yhigashi@hiroshima-u.ac.jp

**Methods**

**Measurements of FMD and NID**

Vascular response to reactive hyperemia in the brachial artery was used for assessment of endothelium-dependent FMD. A high-resolution linear artery transducer was coupled to computer-assisted analysis software (UNEXEF18G, UNEX Co, Nagoya, Japan) that used an automated edge detection system for measurement of brachial artery diameter.^1^ A blood pressure cuff was placed around the forearm. The brachial artery was scanned longitudinally 5-10 cm above the elbow. When the clearest B-mode image of the anterior and posterior intimal interfaces between the lumen and vessel wall was obtained, the transducer was held at the same point throughout the scan by a special probe holder (UNEX Co) to ensure consistency of the image. Depth and gain setting were set to optimize the images of the arterial lumen wall interface. When the tracking gate was placed on the intima, the artery diameter was automatically tracked, and the waveform of diameter changes over the cardiac cycle was displayed in real time using the FMD mode of the tracking system. This allowed the ultrasound images to be optimized at the start of the scan and the transducer position to be adjusted immediately for optimal tracking performance throughout the scan. Pulsed Doppler flow was assessed at baseline and during peak hyperemic flow, which was confirmed to occur within 15 seconds after cuff deflation. Blood flow velocity was calculated from the color Doppler data and was displayed as a waveform in real time. The baseline longitudinal image of the artery was acquired for 30 seconds, and then the blood pressure cuff was inflated to 50 mm Hg above systolic pressure for 5 minutes. The longitudinal image of the artery was recorded continuously until 5 minutes after cuff deflation. Pulsed Doppler velocity signals were obtained for 20 seconds at baseline and for 10 seconds immediately after cuff deflation. Changes in brachial artery diameter were immediately expressed as percentage change relative to the vessel diameter before cuff inflation. FMD was automatically calculated as the percentage change in peak vessel diameter from the baseline value. Percentage of FMD [(Peak diameter - Baseline diameter)/Baseline diameter] was used for analysis. Blood flow volume was calculated by multiplying the Doppler flow velocity (corrected for the angle) by heart rate and vessel cross-sectional area (-r2). Reactive hyperemia was calculated as the maximum percentage increase in flow after cuff deflation compared with baseline flow.

The response to nitroglycerine was used for assessment of endothelium-independent vasodilation. NID was measured as described previously.^1^ Briefly, after acquiring baseline rest images for 30 seconds, a sublingual tablet (75 μg nitroglycerine) was given, and images of the artery was recorded continuously until the dilation reached a plateau after administration of nitroglycerine. Subjects who had received nitrate treatment and subjects in whom the sublingually administered nitroglycerine tablet was not dissolved during the measurement were excluded from this study. NID was automatically calculated as a percent change in peak vessel diameter from the baseline value. Percentage of NID [(Peak diameter - Baseline diameter)/Baseline diameter] was used for analysis. Inter- and intra-coefficients of variation for the brachial artery diameter were 1.6% and 1.4%, respectively, in our laboratory.

**Measurement of Brachial IMT**

Before FMD measurement, baseline longitudinal ultrasonographic images of the brachial artery, obtained at the end of diastole from each of 10 cardiac cycles, were automatically stored on a hard disk for off-line assessment of IMT with a linear, phased-array high-frequency (10-MHz) transducer using an UNEXEF18G ultrasound unit (UNEX Co).^2^ Measurement of IMT was automatically performed on A-mode images of the far wall of the brachial artery. The analysis system automatically chose the measurement point where an image of the posterior intimal interface was clearly obtained. If the measurement point was inappropriate, another clear image site could be manually selected for measurement. A total of 21 points over a 3-mm length of IMT in the 10-mm longitudinal image depicted in the analysis display were measured and the mean value per image was automatically calculated. IMT was measured at the same point in each image. The average of mean values obtained from 10 cardiac cycles was defined as IMT of the brachial artery.

When measuring carotid IMT, we had an anatomical landmark, such as the carotid-artery bulb. Unfortunately, it is difficult to measure the same site of the brachial artery attributable to the lack of an anatomical landmark. Measurement of IMT in the brachial artery was performed at the proper site where the clearest B-mode image of the anterior and posterior intimal interfaces between the lumen and vessel wall was obtained at 5 to 10 cm above the elbow. However, there was little influence of intra- and interpatient variability in the measurement location of the brachial artery at 5 to 10 cm above the elbow in the present study, because the interface on the intima media of brachial artery is relatively smooth and intima-media thickening is not localized or plaques are not presented, resulting in diffuse intima-media thickening. The coefficients of variation of intra- and interobserver brachial IMT measurements were 3.1% and 4.0%, respectively.

**Measurement of baPWV**

Aortic compliance was assessed noninvasively on the basis of Doppler ultrasound measurements of PWV along the descending thoracoabdominal aorta, as previously reported and validated.^3^ Briefly, baPWV, an index of arterial stiffness, was determined by two pressure sensors placed on the right ankle and left brachial arteries to record each pulse wave simultaneously, and the time lag (t) between the notches of the two waves using a pulse wave velocimeter (Form PWV/ABI, model BP-203RPE, Colin Co., Tokyo, Japan). The distance (D) between the two recording sensors was calculated automatically by inputting the value of individual height. The PWV value was calculated as PWV=D/t. PWV was measured for five consecutive pulses, and averages were used for analysis.

**References**

1. Maruhashi T, Soga J, Fujimura N, Idei N, Mikami S, Iwamoto Y, et al. Nitroglycerine-Induced Vasodilation for Assessment of Vascular Function: A Comparison with Flow-Mediated Vasodilation. *Arteriosclerosis, thrombosis, and vascular biology* (2013) 33(6):1401-8. Epub 2013/03/23. doi: 10.1161/atvbaha.112.300934.

2. Iwamoto Y, Maruhashi T, Fujii Y, Idei N, Fujimura N, Mikami S, et al. Intima-Media Thickness of Brachial Artery, Vascular Function, and Cardiovascular Risk Factors. *Arteriosclerosis, thrombosis, and vascular biology* (2012) 32(9):2295-303. Epub 2012/07/17. doi: 10.1161/atvbaha.112.249680.

3. Kimoto E, Shoji T, Shinohara K, Inaba M, Okuno Y, Miki T, et al. Preferential Stiffening of Central over Peripheral Arteries in Type 2 Diabetes. *Diabetes* (2003) 52(2):448-52. Epub 2003/01/24.

**Supplemental Table 1.** Clinical Characteristics of Propensity Score-matched Pairs of Subjects in Model 1

| Variables | Control subjects  (n=131) | Atomic bomb survivors  (n=131) | P value |
| --- | --- | --- | --- |
| Age, year | 76±5 | 76±5 | 1.00 |
| Age at atomic bomb exposure, year |  | 5±4 |  |
| Sex, men/women | 92/39 | 92/39 | 1.00 |
| Body mass index, kg/m^2^ | 23.0±3.6 | 23.5±3.0 | 0.17 |
| Systolic blood pressure, mmHg | 129±19 | 128±19 | 0.81 |
| Diastolic blood pressure, mmHg | 74±11 | 73±11 | 0.28 |
| Heart rate, bpm | 69±11 | 66±11 | 0.01 |
| Total cholesterol, mg/dL | 185±38 | 178±36 | 0.11 |
| Triglycerides, mg/dL | 121±65 | 120±70 | 0.94 |
| HDL cholesterol, mg/dL | 59±18 | 59±18 | 0.92 |
| LDL cholesterol, mg/dL | 107±33 | 99±29 | 0.05 |
| Glucose, mg/dL | 114±31 | 113±27 | 0.87 |
| HbA1c, % | 6.0±0.6 | 6.2±0.8 | 0.07 |
| eGFR, mL/min per 1.73 m^2^ | 59.4±19.0 | 56.0±18.8 | 0.16 |
| High-sensitivity CRP, mg/dL | 0.32±0.63 | 0.36±0.51 | 0.81 |
| Medical history, n (%) |  |  |  |
| Hypertension | 105 (80.2) | 102 (77.9) | 0.65 |
| Dyslipidemia | 79 (60.3) | 81 (61.8) | 0.80 |
| Diabetes mellitus | 41 (31.3) | 49 (37.4) | 0.30 |
| Previous coronary heart disease | 26 (19.9) | 34 (26.0) | 0.24 |
| Previous stroke | 11 (8.4) | 17 (13.0) | 0.23 |
| Current smoker, n (%) | 14 (10.7) | 10 (7.6) | 0.39 |
| Medication, n (%) |  |  |  |
| Antihypertensive drugs | 100 (76.3) | 107 (81.7) | 0.29 |
| Lipid-lowering drugs | 57 (43.5) | 72 (55.0) | 0.06 |
| Antidiabetic drugs | 29 (22.1) | 36 (27.5) | 0.62 |

HDL indicates high-density lipoprotein; LDL, low-density lipoprotein; HbA1c, hemoglobin A1c; eGFR, estimated glomerular filtration rate; CRP, C-reactive protein.

Results are presented as means±SD for continuous variables and percentages for categorical variables.

**Supplemental Table 2.** Clinical Characteristics of Propensity Score-matched Pairs of Subjects in Model 2

| Variables | Control subjects  (n=119) | Atomic bomb survivors  (n=119) | P value |
| --- | --- | --- | --- |
| Age, year | 75±5 | 76±5 | 0.67 |
| Age at atomic bomb exposure, year |  | 5±4 |  |
| Sex, men/women | 84/35 | 83/36 | 0.89 |
| Body mass index, kg/m^2^ | 23.8±3.2 | 23.4±2.8 | 0.37 |
| Systolic blood pressure, mmHg | 130±17 | 128±19 | 0.66 |
| Diastolic blood pressure, mmHg | 73±10 | 73±11 | 0.70 |
| Heart rate, bpm | 68±11 | 67±11 | 0.63 |
| Total cholesterol, mg/dL | 180±31 | 178±37 | 0.69 |
| Triglycerides, mg/dL | 123±81 | 121±71 | 0.83 |
| HDL cholesterol, mg/dL | 57±15 | 58±18 | 0.69 |
| LDL cholesterol, mg/dL | 101±25 | 100±30 | 0.61 |
| Glucose, mg/dL | 118±34 | 114±28 | 0.27 |
| HbA1c, % | 6.1±0.7 | 6.2±0.8 | 0.31 |
| eGFR, mL/min per 1.73 m^2^ | 60.8±18.5 | 56.3±19.1 | 0.07 |
| High-sensitivity CRP, mg/dL | 0.38±0.73 | 0.37±0.52 | 0.93 |
| Medical history, n (%) |  |  |  |
| Hypertension | 96 (80.7) | 94 (79.0) | 0.75 |
| Dyslipidemia | 77 (64.7) | 75 (63.0) | 0.79 |
| Diabetes mellitus | 51 (42.9) | 47 (39.5) | 0.60 |
| Previous coronary heart disease | 32 (27.1) | 33 (27.7) | 0.91 |
| Previous stroke | 21 (17.7) | 16 (13.5) | 0.37 |
| Current smoker, n (%) | 6 (5.0) | 10 (8.4) | 0.30 |
| Medication, n (%) |  |  |  |
| Antihypertensive drugs | 94 (79.0) | 99 (83.2) | 0.41 |
| Lipid-lowering drugs | 61 (51.3) | 68 (57.1) | 0.36 |
| Antidiabetic drugs | 40 (33.6) | 35 (29.4) | 0.49 |

HDL indicates high-density lipoprotein; LDL, low-density lipoprotein; HbA1c, hemoglobin A1c; eGFR, estimated glomerular filtration rate; CRP, C-reactive protein.

Results are presented as means±SD for continuous variables and percentages for categorical variables.

**Supplemental Table 3.** Clinical Characteristics of the Subjects Corresponding to Study Protocol 2

| Variables | Atomic bomb survivors  (n=10) |
| --- | --- |
| Age, year | 75±4 |
| Age at atomic bomb exposure, year | 5±4 |
| Sex, men/women | 7/3 |
| Body mass index, kg/m^2^ | 23.9±2.6 |
| Systolic blood pressure, mmHg | 121±17 |
| Diastolic blood pressure, mmHg | 72±13 |
| Heart rate, bpm | 65±5 |
| Total cholesterol, mg/dL | 178±28 |
| Triglycerides, mg/dL | 131±69 |
| HDL cholesterol, mg/dL | 61±20 |
| LDL cholesterol, mg/dL | 95±25 |
| Glucose, mg/dL | 118±40 |
| HbA1c, % | 6.2±0.5 |
| eGFR, mL/min per 1.73 m^2^ | 54.3±18.6 |
| Medical history, n (%) |  |
| Hypertension | 9 (90.0) |
| Dyslipidemia | 8 (80.0) |
| Diabetes mellitus | 3 (30.0) |
| Previous coronary heart disease | 4 (40.0) |
| Previous stroke | 3 (30.0) |
| Current smoker, n (%) | 1 (1.0) |
| Medication, n (%) |  |
| Antihypertensive drugs | 9 (90.0) |
| Lipid-lowering drugs | 8 (80.0) |
| Antidiabetic drugs | 3 (30.0) |
| FMD, % | 3.9±1.5 |
| NID, % | 11.8±4.4 |
| baPWV, cm/s | 1630±234 |
| Brachial artery IMT, mm | 0.32±0.03 |
| Radiation dose, Gy | 0.23±0.18 |

HDL indicates high-density lipoprotein; LDL, low-density lipoprotein; HbA1c, hemoglobin A1c; eGFR, estimated glomerular filtration rate; FMD, flow-mediated vasodilation; NID, nitroglycerine-induced vasodilation; baPWV, brachial-ankle pulse wave velocity; IMT, intima-media thickness. Results are presented as means±SD for continuous variables and percentages for categorical variables.

**Supplemental Figure 1**


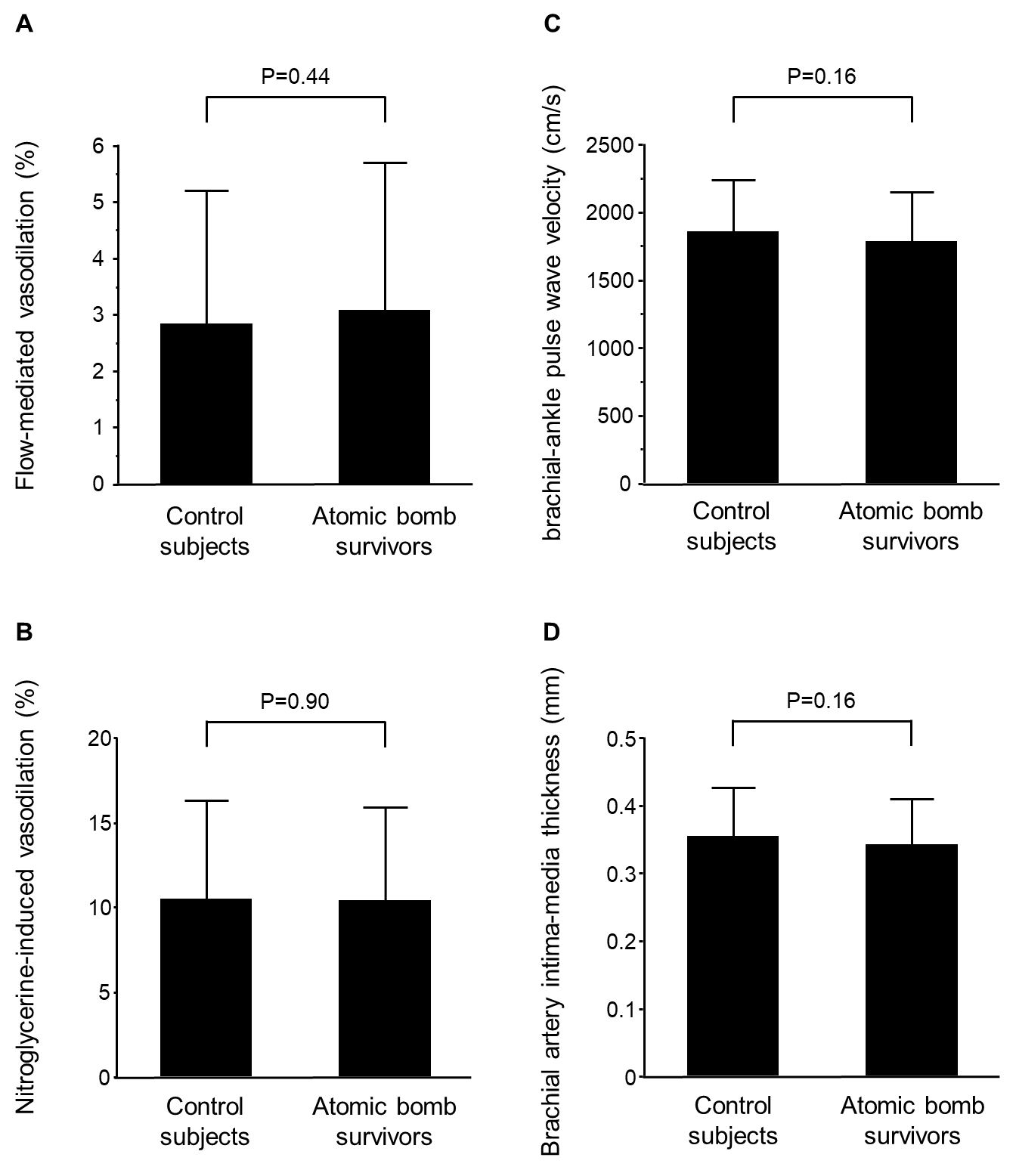


**Supplemental Figure 1.** Bar graphs show flow-mediated vasodilation (A), nitroglycerine-induced vasodilation (B), brachial-ankle pulse wave velocity (C) and brachial artery intima-media thickness (D) in control subjects and atomic bomb survivors of propensity score-matched pairs in model 1.

**Supplemental Figure 2**


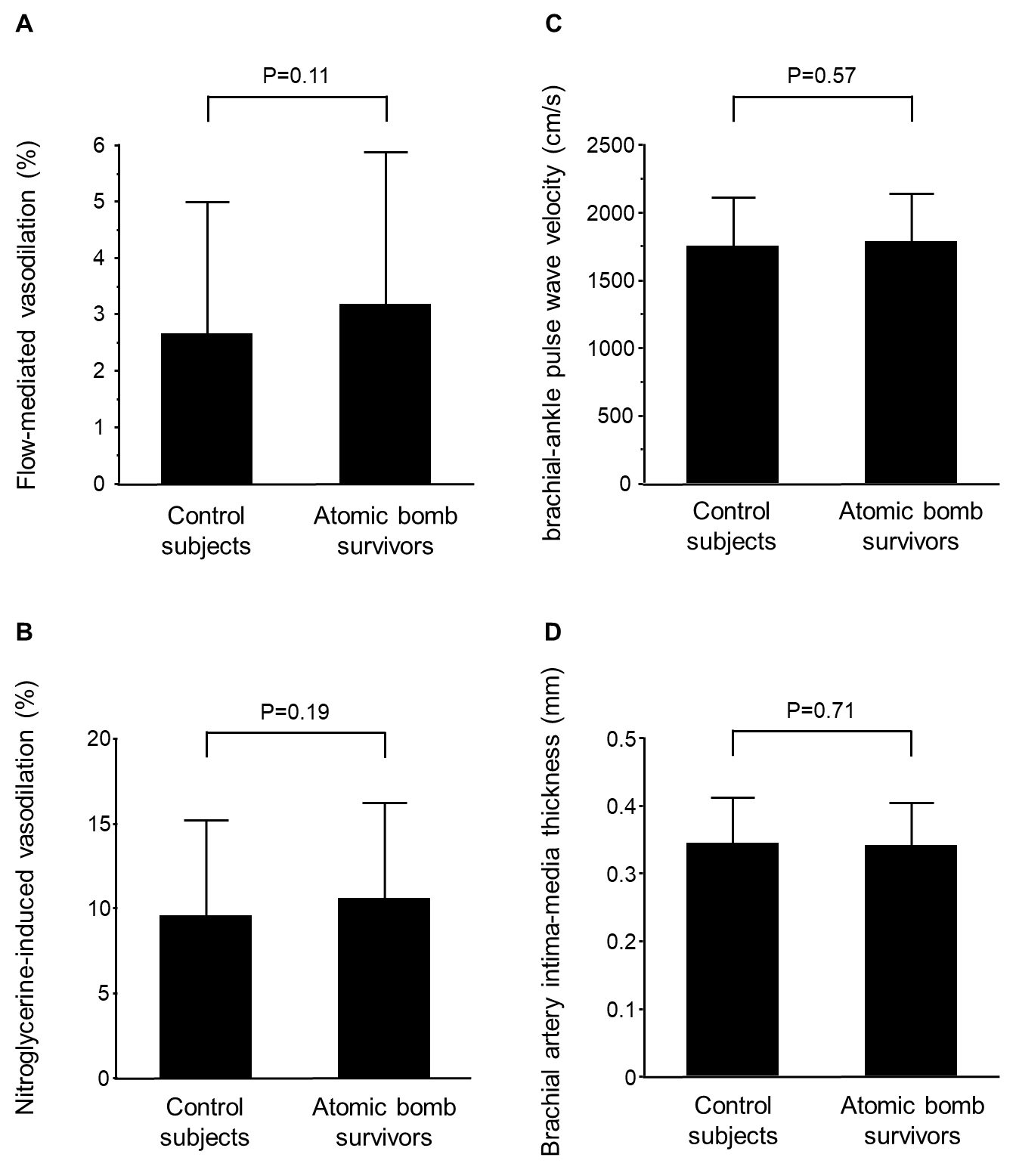


**Supplemental Figure 2.** Bar graphs show flow-mediated vasodilation (A), nitroglycerine-induced vasodilation (B), brachial-ankle pulse wave velocity (C) and brachial artery intima-media thickness (D) in control subjects and atomic bomb survivors of propensity score-matched pairs in model 2.
